# Response to ‘The implications of three major new trials for the effect of water, sanitation and hygiene on childhood diarrhea and stunting: a consensus statement’ by Cumming et al.

**DOI:** 10.1186/s12916-019-1414-6

**Published:** 2019-09-26

**Authors:** Megan Wilson-Jones, Kyla Smith, Dan Jones, Helen Hamilton, Leah Richardson, Alison Macintyre, Om Prasad Gautam, Erik Harvey, Henry Northover

**Affiliations:** 1 WaterAid UK, 47–49 Durham St, Vauxhall, London, SE11 5JD UK; 2 WaterAid Sweden, Hannebergsgatan 33, 171 68 Solna, Sweden; 3 WaterAid Australia, Level 9, 176 Wellington Parade, East Melbourne, VIC 3002 Australia

**Keywords:** Water, Sanitation, Hygiene, Diarrhea, Undernutrition, Stunting

## Abstract

Please see related article: https://doi.org/10.1186/s12916-019-1410-x

## Background

We welcome the thoughtful analysis in the statement by Oliver Cumming, et al. (2019) [1] regarding the latest evidence linking water, sanitation and hygiene (WASH) interventions and childhood stunting and diarrhea. First, we congratulate the study teams involved in WASH-Benefits [2, 3] and SHINE [4] for the delivery of these unprecedented large-scale, high-quality trials. The trials significantly add to the evidence available on this topic, and represent an important contribution to the WASH sector in recent years.

## WaterAid: using the evidence to develop ‘transformative’ WASH

These studies further highlight the complexity of the relationship between WASH and undernutrition, and reinforce the need for detailed consideration of the results – in the context of the larger body of evidence – to inform future policies and programs. As WASH practitioners, we at WaterAid are engaging with the findings of these studies to better understand the implications for our work. At the same time, we acknowledge our role in contributing to building operational experience and knowledge to fill critical evidence and programmatic gaps that these types of studies are not designed to answer. Our focus is on working towards the achievement of universal access to WASH for everyone, everywhere, and on maximizing the contribution of WASH to achieving the health and nutrition-related Sustainable Development Goals (SDGs), among others.

As stated by the authors of the consensus note, we must acknowledge that large-scale improvements in WASH have been important drivers of public health for centuries. This is in addition to the host of other benefits associated with increasing access to WASH, such as time-savings, dignity, and quality of life and safety, particularly of women and children. We also acknowledge that these improvements have come as a result of important, large-scale transformations of water and sanitation systems, and substantial changes in hygiene behaviors, which extend beyond the implementation of basic, localized WASH interventions. This is the scale at which the ambitious SDGs call for, including safely managed sanitation and piped water supplies in reach of everyone.

WaterAid supports the notion of more comprehensive interventions. Broader integrated development strategies are needed across key sectors, such as WASH, health, nutrition, education and housing, which are all fundamental in underpinning wider societal and economic development and, consequentially, healthy and productive populations. Such strategies need to be layered and iterative over time. We argue that WASH interventions themselves may need to be layered and iterative to achieve universal access, ensuring that no one is left behind. Such interventions must focus on strengthening systems to sustain these services and behaviors. This is where WaterAid and other actors are currently focusing their efforts.

Based on our programmatic experience to date, and that of others, we hope to further contribute to this discussion in the following ways:

### Blocking multiple routes of transmission with high coverage interventions

Given the complexity of the relationship between WASH and undernutrition, and the multitude of possible transmission routes, the WASH, health and nutrition sectors must continue to work together to identify other components that are likely to be important for improving nutritional outcomes. These components include food hygiene [5]; provision of a continuous, safe water supply; community-wide sanitation coverage [6]; environmental hygiene (including protection from animal feces); and institutional and community-wide WASH access and activities (to also address settings when children are not at home).

### Behavior change

We recognize the complexities of sustainably changing and sustaining WASH and nutrition-related behaviors in any setting, and the important work that academics and implementers are doing in this space. Increased collaboration is needed to share successes and learning of innovative, practical and evidence-based behavior change interventions that move beyond increasing knowledge. We at WaterAid can contribute to this area of work by sharing our experiences from multiple countries.

### Systems strengthening and cross-sectoral working

We acknowledge that interventions like those designed for the WASH and SHINE studies exist. However, WaterAid and other organizations are moving away from siloed and project-based approaches, to working with governments and partners to plan and deliver sustainable WASH programs that proactively strengthen government and community systems. We are also coordinating with other sectors, including health, education, and nutrition. As a sector, we need to build understanding on how this can be done in different contexts, and how we can reach those who are left behind and not accessing services. WaterAid will continue to build its experience of capturing and sharing evidence and practical lessons on the integration of WASH and other sectors at the policy, institutional coordination, and programmatic implementation levels. By understanding potential entry points for effective integration, and context-specific actions that decision-makers can take to support this, we can create an environment that is more conducive to cross-sector working. For example, we have worked with the World Health Organization to support the development of a Neglected Tropical Diseases Toolkit [7] to strengthen collaboration between WASH and health programs. In Nepal, we are working with the government to integrate hygiene promotion into the national routine immunization systems at scale [8].

### Evidence and research

We seek further discussion and engagement with the academic community to strengthen implementation research on defining and delivering ‘transformative’ WASH programs that focus on strengthening systems needed to sustain inclusive, long-lasting access to WASH and behavior change. We also seek to understand how best to work with the health and nutrition sectors to maximize mutual benefits.

## Conclusions

We hope this discussion will continue with close collaboration between academics, non-governmental organizations, governments, donors and the private sector. In doing so, we will move towards the practical definition and design of ‘transformative’ WASH that is supported by a strong evidence base so that everyone, everywhere, has access to WASH.

## Data Availability

Not applicable.
